# Comprehensive management of frailty. A broader perspective of implementation project “**Systemic Approach to Frailty with a Focus on Pre-Frailty for Healthy and High-Quality Aging**”

**DOI:** 10.1016/j.tjfa.2025.100075

**Published:** 2025-08-05

**Authors:** Branko Gabrovec, Nadja Cirar, Katarina Cesar, Rade Pribakovič Brinovec, Matej Vinko, Nina Pirnat, Urška Erklavec, Hajdi Kosednar, Jernej Bevk, Ivan Eržen, Tajda Golja, Anja Jutraž

**Affiliations:** a National Institute of Public Health, Slovenia; b Ministry of Health, Slovenia; c University of Maribor, Faculty of Health Sciences, Slovenia; d University of Ljubljana, Medical Faculty, Slovenia

**Keywords:** Frailty, Aging, Management, Comprehensive, Healthy living environment

## Abstract

Managing pre-frailty and frailty is crucial for maintaining the health and independence of older adults. Through **“**Systemic Approach to Frailty with a Focus on Pre-Frailty for Healthy and High-Quality Aging” project, Slovenia is establishing a systematic approach to identify and address frailty, while developing solutions to support preventive measures across healthcare and community levels. Key steps include integrating health and social care systems, strengthening health literacy, adapting and co-creating healthy living environments through a community-based approach and social participation, all essential for promoting healthier and higher-quality aging in the population.

This paper outlines the innovative conceptual and methodological approach to project development and its anticipated outcomes.

## Highlights

• Managing pre-frailty and frailty is crucial for maintaining the health and independence of older adults.
• Systemic Approach to Frailty with a Focus on Pre-Frailty for Healthy and High-Quality Aging” project.
• Paper outlines the innovative conceptual and methodological approach to project development and its anticipated outcomes.

## 1 Introduction

Frailty has been described as a distinctive health state related to the ageing process, in which multiple body systems gradually lose their in-built reserves [[1], [2], [3], [4], [5]]. There are two operational definitions of frailty in common use, within which frailty estimates have lower variation [6]: frailty phenotype and frailty index [5].

The frailty phenotype, proposed by Fried et al. [7], describes a group of individual characteristics (weakness, slowness, low level of physical activity, exhaustion, and unintentional weight loss) that predict poor outcomes. A person is judged to be frail if they have at least three of the five characteristics; pre-frail if they have one or two characteristics; and robust if they have none of the characteristics. The frailty index, or cumulative deficit model, was developed by Mitnitski et al. [8] In this model, people accumulate ‘deficits’ over time that increase their risk of poor outcomes. Frailty indices have been shown to be associated with mortality, hospitalization and nursing home admission [9].

In Slovenia life expectancy at birth for women in 2025 is 85, men 79,1 years [10]. Leading cause of death: men (35 % cancer, 30 % cardiovascular disease), women (43 % cardiovascular disease, 27 % cancer) [11]. Although Slovenian residents have a longer life expectancy, they experience poorer health outcomes, including malnutrition, a sedentary lifestyle, sarcopenia, chronic non-communicable diseases, polypharmacy, poor oral health, and frailty [[12], [13], [14], [15], [16], [17], [18]].

Research shows that age-standardized among older adults (≥65 years), prevalence (95 % CI) of frailty and pre-frailty in Slovenia were 14.9 % (13.3–16.5) and 42.5 % (39.8–45.2), respectively [19].

Since healthcare expenditures for older individuals are significantly higher than for younger populations, demographic aging contributes to rising costs. This makes both the fiscal sustainability of healthcare systems and equitable access to services increasingly challenging. Frailty-specific data reveals particularly stark disparities: frail individuals consume up to 469 % more healthcare resources than their robust peers [20]. Slovenia has already committed to strengthening holistic population health approaches through its long-term strategy - the Resolution on the National Healthcare Plan 2016–2025 'Together for a Healthy Society'. This includes specific measures to address frailty prevention and management.

### 1.1 Advantage joint action and project “Systemic approach to frailty with a focus on pre-frailty for healthy and high-quality aging”

The European Commission and 22 European Union Member States (EU MSs) cofounded the first Joint Action (JA) in frailty: ADVANTAGE (2017 – 2019). It aimed to build a common framework to push frailty as a public health priority contributing to a homogeneous and evidence-based approach across Europe. This has been achieved through the following steps [21,22]:

• Reaching a common understanding of frailty and its associated factors, in order to promote the required and sustainable changes in the organization and implementation of care in the health and social systems of EU MSs;
• Sharing a common European framework for screening, early diagnosis, prevention, assessment and management of frailty;
• Developing a common strategy for frailty prevention and management (Frailty Prevention Approach), including raising awareness and advocacy among stakeholders, especially policy- and decision-makers.

As an active partner in the Advantage Joint Action, Slovenia led a work package focused on individual-level frailty management. The results informed the development of a national implementation project that will address frailty systematically and comprehensively. Launching in 2025, this project aims to establish a systemic approach for frailty prevention and management, with a special emphasis on healthy aging and the co-creation of healthy living environments.

This paper describes a novel approach to planning and designing the implementation of systematic, comprehensive frailty management.

## 2 Methodology

The project content was developed through **1)** Foundations and recommendations from the Advantage Joint Action (Slovenia Roadmap) and European Guide for Management of Frailty at Individual Level Including Recommendations and Roadmap, **2)** Member States Survey, **3)** Good practices and literature review and **4)** Expanding the Frailty Framework: mental, social, and environmental dimensions.

### 2.1 Foundations and recommendations from the advantage joint action (Slovenia roadmap) and European guide for management of frailty at individual level including recommendations and roadmap

Widespread evidence shows that frailty emergence or its onset can be delayed through intervention in the early stages. Recommendations and Roadmap targeted six key fields of intervention that must be taken into consideration while tackling frailty: prevention, clinical management, nutrition, physical exercise, drugs, and information and communication technologies (ICTs).

The prevention of frailty should include both the promotion of healthy lifestyle among middle-aged and older people and service organisations, and an emphasis on enablement and maintaining independence:

• To tackle frailty, we should take steps to raise greater awareness of empowerment of older people who have a strong aversion to the term “frail”.
• Nutritional intervention is proposed widely to be an important component of frailty management, while inadequate nutritional intake is an important modifiable risk factor for frailty.
• Sedentary lifestyle is a risk factor for developing frailty. Exercise can improve physical performance and reduce physical frailty. Exercise in older people with frailty is effective and relatively safe, and may reverse frailty.
• Many tools are available to assess polypharmacy, but none address all aspects of appropriate polypharmacy. The various aspects to be considered include multimorbidity, safety, efficacy and acceptability of medicines, the patient’s wellbeing, social circumstances and outcomes.
• ICT services have gained increasing attention for dealing with people with frailty due to the development of solutions for tele-monitoring (to monitor health status at distance) and tele-treatment (to work on the functional status). Results of these reviews suggest that the acceptance and employment of these new technologies remains problematic, especially for older people [23,24], although recent studies have shown promising results [25].

Recommendations from the Advantage Joint Action were prioritized through a dual feasibility-impact assessment, with nutrition interventions, physical activity programs, and polypharmacy management selected for Slovenian pilot implementation based on local risk factor prevalence. The process incorporated evidence-based guidelines developed using GRADE methodology**.**

### 2.2 Members states survey

A survey of Member States (MS) was conducted in the Joint Action ADVANTAGE MS to access policies, strategies, programmes, guidelines and interventions regarding countries’ specific frailty situations. The MS’ survey was conducted from January to March 2018.

The study of the background frailty situation reports made clear that the classification of the MS should be organized in five very general levels:

• Sustainable: There is an evaluated national strategy or there is an agreed plan to sustain it;
• Advanced: There is a national strategy on that item;
• Well-developed: Relevant interventions/programmes are being carried out in many parts of the MS;
• Fair: Something is being done in some places in the MS;
• Basic: Nothing is going on in the MS in relation to that item.

The classifications consisting of six variables (Prevention, Clinical Management, Nutrition, Physical Activity, Drugs, and Information and Communication Technology) are presented in Table 1.

**Table 1:** Member States classifications according to level of implementation [22].

**Implementation level**	**EU Member States that achieve a certain level of implementation in each area**					
	**Prevention**	**Clinical management**	**Nutrition**	**Physical activity**	**Drugs**	Information and communication technology
**Sustainable**		Belgium, Italy, Great Britain				
**Advanced**	Finland, France, Italy, Netherland, Poland, Spain, Great Britain	France, Ireland	Finland, France, Greece, Poland, Germany	Finland	Belgium, Finland, France, Spain, Great Britain	
**Well developed**		Spain	Belgium, Ireland, Netherland, Spain, Great Britain	France, Ireland, Italy, Malta, Portugal, Spain, Great Britain	Ireland, Italy, Malta, Germany, Portugal, **Slovenia**	Finland, France, Italy, Great Britain
**Appropriate**	Austria, Belgium, Bulgaria, Cyprus, Greece, Ireland, Hungary, Germany, Portugal	Bulgaria, Austria, Malta, Germany	Austria, Cyprus, Italy, Lithuania, Portugal, **Slovenia**	Austria, Belgium, Greece, Germany, Netherland, Poland, Romania, **Slovenia**	Austria, Cyprus, Greece, Lithuania, Poland, Romania	Austria, Belgium, Greece, Germany, Portugal, Romania, **Slovenia**, Spain
**Basic**	Croatia, Lithuania, Malta, Romania, **Slovenia**	Cyprus, Greece, Finland, Croatia, Hungary, Lithuania, Netherland, Poland, Portugal, Romania, **Slovenia**	Bulgaria, Croatia, Hungary, Malta, Romania	Bulgaria, Cyprus, Croatia, Lithuania	Bulgaria, Croatia, Hungary, Netherland	Bulgaria, Cyprus, Croatia, Hungary, Ireland, Lithuania, Malta, Netherland, Poland
**Number of measures in the three highest levels of implementation**	7	6	10	7	11	4

### 2.3 Good practices and literature review

The collection of good practices was opportunistic, rather than systematic. It was based on the former EU-funded programmes, European Innovation Partnership on Active and Healthy Ageing (EIP-AHA) and Joint Action on Chronic Diseases and Promoting Healthy Ageing Across the Life Cycle (JA CHRODIS), and key stakeholders and national policy documents known by partners.

A PRISMA-guided systematic review (PubMed, Cochrane, Embase, UpToDate, CINAHL) examined six domains: prevention, clinical management, nutrition, physical activity, medication management, and ICT. Evidence was appraised using GRADE methodology.

In total, for the task Prevention, 391,910 search results were identified and 31 articles/sources included in the analysis; for the task Clinical management, 67,432 search results were identified and 27 articles/sources included in the analysis; for the task Nutrition, 39,885 search results were identified and 28 articles/ sources included in the analysis; for the task Physical activity, 620,043 search results were identified and 25 articles/sources included in the analysis; for the task Drugs, 28,796 search results were identified and 25 articles/sources included in the analysis; for the task ICTs, 124,634 search results were identified and 33 articles/sources included in the analysis.

### 2.4 Expanding the frailty framework: mental, social, and environmental dimensions

To ensure a broader and more comprehensive approach to frailty beyond physical aspects, we also addressed mental and social frailty. It is important to emphasize that mental and social frailty are not synonymous with mental disorders. Rather, they represent a broader concept that includes individuals who may not meet diagnostic criteria for mental illness but are nevertheless at higher risk of developing psychological difficulties and declining health. These two dimensions of frailty allow for a better understanding of vulnerability in older adults and more targeted interventions to improve their health and well-being. By early identification and management of mental and social frailty, we can prevent or at least mitigate the development of more serious issues, enabling older adults to achieve healthier and more active aging.

Mental frailty is discussed in the literature as a complex syndrome encompassing psychological and cognitive aspects and is often used interchangeably with the term psychological frailty [26]. Social frailty, on the other hand, is often associated with feelings of loneliness, lack of social support, and limited social engagement [27].

Additionally, age-friendly environments and communities were also addressed, consisting of built, living environments, local communities, safety measures, and the inclusion of non-governmental organization (NGOs) to promote and sustain social participation in line with the resolution endorsed by the World Health Assembly in May 2024 [28]. The Living Environment Assessment Tool developed under the JAHEE project was reviewed for its potential to support frailty prevention and management. The tool includes both physical aspects (such as transport, parking, and the natural environment) and social factors (such as opportunities for interaction among residents) and helps local communities, decision-makers and other stakeholders to identify opportunities to improve their environments in order to promote health, well-being, and quality of life [29].

The proposed framework was further shaped through insights from multiple EU joint initiatives, notably CHRODIS, CHRODIS-PLUS, JACARDI, JA PreventNCD [30,31].

## 3 Results

The foundations and recommendations derived from the Advantage Joint Action project, along with the expansion of variables that influence and/or have positive effects on the identification, prevention, and management of frailty, contributed to an innovative and comprehensive proposal for the implementation project in Slovenia.

The primary objective of the project is to propose the establishment of a systemic approach for the prevention and management of frailty, prioritizing healthy aging and quality of life in older adults.

Proposed specific project objectives:

• Analysis, development, pilot testing, and evaluation of interventions aimed at strengthening health and managing risk factors in pre-frail/frail individuals, as well as developing models for assessing physical pre-frailty and frailty.
• Raising awareness and improving understanding of the role of physical and mental health in pre-frailty and frailty, with a focus on preventive measures to maintain physical independence. This includes emphasizing psychological and social aspects in broader society and enhancing social inclusion among older adults as a key factor in promoting mental well-being.
• Co-creating age-friendly community guidelines (e.g., walkability audits, social hub design) with NGOs and local governments, aiming for 30 % adoption in pilot municipalities by 2027.
• Designing and adapting a digital health platform to improve digital and health literacy, tailored for all healthcare stakeholders.
• Implementing a tiered digital health platform (e.g., tele-monitoring for high-risk cases, literacy apps for pre-frail adults) targeting 50 % uptake among primary care providers.

**Fig. 1:**
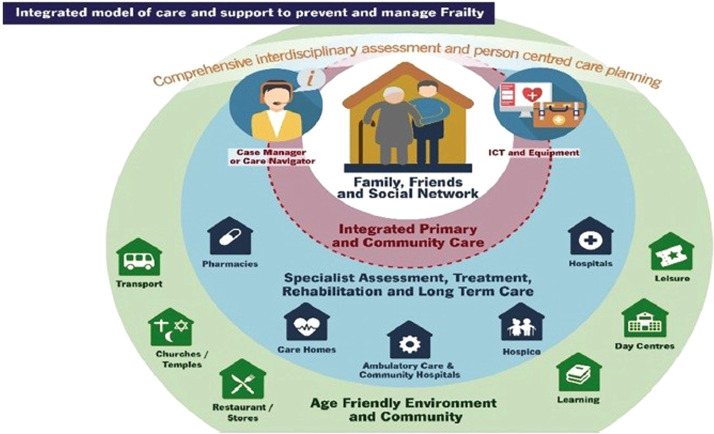
Integrated model of care and support to prevent and manage frailty [4].

Our proposal conceptually and innovatively builds upon the 'Integrated Model of Care and Support to Prevent and Manage Frailty' from the Advantage Joint Action project, representing a new systematic approach to frailty management (Fig. 1).

The project proposal enhances the integrated model by incorporating a comprehensive approach to frailty, including mental health and social frailty components, while also expanding the role of ICT solutions, safety measures, co-creation of healthy living environments and NGOs. The project work packages include not only substantive content but also clearly measurable objectives and indicators, along with well-defined risk assessments.

The proposal received approval from the Ministry of Health and the Ministry of Cohesion and Regional Development, with implementation scheduled from spring 2025 through December 2028.

The project implementation must incorporate monitoring mechanisms and systemic organization to ensure sustainability post-project. Consequently, the Ministry of Health is involved from the initial phase to facilitate implementation frameworks and foster multi-sectoral cooperation among government entities, professional bodies, civil society organizations, social partners, and other relevant stakeholders.

## 4 Conclusion

Managing pre-frailty and frailty is crucial for maintaining the health and independence of older adults. Through "Frailty Management" project, Slovenia is establishing a systematic approach to identify and address pre-frailty, while developing solutions to support preventive measures across healthcare and community levels. Key steps include integrating health and social care systems, strengthening digital and health literacy, and adapting living environments, all essential for promoting healthier and higher-quality aging in the population.

Methodologically**,** the project builds on insights, proposals, and recommendations from prior initiatives, particularly the Advantage Joint Action*.* Its conceptual innovation lies in a comprehensive frailty management framework that incorporates physical health, mental health and social frailty considerations, environmental and local community perspectives, ICT tools, the role of NGOs (social participation), safety measures, education and research.

The project also serves as a platform for innovative approaches in mental and social health, environmental components, and social participation. Population aging drives broader societal transformations, necessitating comprehensive, multidisciplinary responses - as exemplified by our frailty prevention and management initiatives.

## Limitations

The project faces operational limitations regarding sustainable multi-stakeholder participation and availability of adequately skilled human resources.

## Funding

The operation is implemented under the European Cohesion Policy Programme 2021-2027 in Slovenia, Priority 7: "Long-term Care and Health, and Social Inclusion", Specific Objective ESF+ 4.11. The investment is co-financed by the EU through the European Social Fund Plus (ESF+) and the Republic of Slovenia's State Budget as the national contribution.

## Declaration of competing interest

The authors declare that they have no known competing financial interests or personal relationships that could have appeared to influence the work reported in this paper.
